# Inhibition of the Progesterone Nuclear Receptor during the Bone Linear Growth Phase Increases Peak Bone Mass in Female Mice

**DOI:** 10.1371/journal.pone.0011410

**Published:** 2010-07-01

**Authors:** Wei Yao, Weiwei Dai, Mohammad Shahnazari, Aaron Pham, Zhiqiang Chen, Haiyan Chen, Min Guan, Nancy E. Lane

**Affiliations:** Department of Internal Medicine, Center for Healthy Aging, University of California Davis Medical Center, Sacramento, California, United States of America; University of Las Palmas de Gran Canaria, Spain

## Abstract

Augmentation of the peak bone mass (PBM) may be one of the most effective interventions to reduce the risk of developing osteoporosis later in life; however treatments to augment PBM are currently limited. Our study evaluated whether a greater PBM could be achieved either in the progesterone nuclear receptor knockout mice (PRKO) or by using a nuclear progesterone receptor (nPR) antagonist, RU486 in mice. Compared to their wild type (WT) littermates the female PRKO mice developed significantly higher cancellous and cortical mass in the distal femurs, and this was associated with increased bone formation. The high bone mass phenotype was partially reproduced by administering RU486 in female WT mice from 1–3 months of age. Our results suggest that the inhibition of the nPR during the rapid bone growth period (1–3 months) increases osteogenesis, which results in acquisition of higher bone mass. Our findings suggest a crucial role for progesterone signaling in bone acquisition and inhibition of the nPR as a novel approach to augment bone mass, which may have the potential to reduce the burden of osteoporosis.

## Introduction

Osteoporosis is a major public health problem that currently affects 44 million Americans. Approximately one of every two women and one of every four men will suffer a fracture due to osteoporosis during their lifetimes. Eighty percent of bone density is genetically determined while the other 20% is determined by lifestyle and environmental factors such as diet, exercise, smoking, and various medications [1]. The two most significant risk factors associated with the development of osteoporosis are the peak bone mass achieved and the rate of bone loss. Peak bone mass is dependent on the rate of bone growth, which is highest during infancy and during the pubertal growth spurt. Adolescence is a particularly critical period of bone acquisition, since the rate of bone growth is nearly double that from earlier years, and approximately 40% of the peak bone mass is acquired from periosteal expansion. At the end of puberty, the epiphyseal growth plates fuse and linear bone growth ends. However, bone mass continues to increase both at the endocortical and trabecular bone surfaces and within a few years of the age of 20, 90–95% of the peak bone mass has developed [2]. Although the intake of calcium and vitamin D through diet and supplements and weight-bearing exercise during puberty have modest impacts on the augmentation of peak bone mass [3], [4], [5], [6], interventions with greater efficacies have yet to be developed.

Progesterone is known for its effects on the reproductive system, and its physiological roles in skeletal metabolism remains unclear. In clinical studies, oral contraceptives that contained progesterone [7], [8] resulted in a modest reduction of bone mineral density (BMD) that was within one standard deviation of placebo-treated controls in both the central and peripheral skeleton [9], [10], [11], [12], [13], [14], [15]. In postmenopausal women, treatment with a synthetic progestin (norethisterone) did not prevent bone loss [16], [17]. In contrast, treatment with cyclic medroxyprogesterone increased spinal cancellous bone density by approximately 1.7% during a one-year long, randomized, double-blind, placebo-controlled trial in premenopausal women with disturbed menstruation [18]. In animal models, reports of progesterone's effects on bone density have been variable and are influenced by estrogen, the dose of progesterone administered, skeletal site analyzed, and the stage of skeletal maturation [19], [20].

Progesterone nuclear receptors (nPR) are present in human osteoblasts [21], [22], [23] and osteoclasts [24]. A high cancellous bone mass phenotype was reported in female progesterone receptor knockout mice (PRKO) in the proximal tibia metaphysis at 26 weeks of age [25]. The investigators reported that a higher bone mass was associated with higher surface-based bone formation rates that were assessed in 24-week-old PRKO mice compared to control animals.

Interestingly, the nPR antagonist, RU486, administered at a dose of 10 mg/kg for four weeks prevented bone loss in three-month-old estrogen-deficient rats [26]. However, another study reported that RU486 did not stimulate bone formation when used at the same dose in normal, estrogen-intact, sexually mature three-month-old rats [27]. Based on these data, we hypothesized that the “timing” of the progesterone receptor' inhibition is critical for augmenting bone mass. We found that compared to the WT littermates, female mice lacking nPR (PRKO) had accelerated bone formation and cancellous bone gain in the distal femoral metaphysis between 1–3 months of age, and the cancellous bone mass was maintained thereafter. In contrast, the male PRKO mice and WT littermates had similar bone acquisition from one to six months of age, but the PRKO male mice had less bone resorption and less age-related bone loss compared to WT littermates from 6–12 months of age. RU486, administered immediately after weaning in one to three-month old female WT mice recapitulated the rapid gain in femoral bone mass and the high bone mass phenotype. Our findings illustrate that the nPR inhibit bone acquisition and bone formation in female mice; and that the temporary inhibition of nPR during the linear bone growth period may provide a novel approach to augment peak bone mass.

## Results

Bones from female and male PRKO mice and WT littermates (C57/BL6 backgrounds) were analyzed from 4–48 weeks (1–12 months) of age. The first evaluation was performed at four weeks of age (one month) which is pre-pubertal time point that is just prior to rapid skeletal acquisition. The second evaluation was performed at 12 weeks of age (three months), which is a post-pubertal time point when the peak bone mass has been mostly achieved, bone mass acquisition has slowed, and the mouse reproductive system is fully developed. The evaluation was repeated every three months until the mice were 12 months old to record longitudinal and cortical bone growth. We also performed cell culture experiments using 12-week-old, mice when the maximum bone growth was observed.

### Body Weights

Body weights were recorded each month. The body weight increased with age from 15 grams to 26 grams from four to 24 weeks of age. There were no differences in body weight between the genotypes.

### Gonadal hormones or reproductive hormones

Gonadal hormone levels have been reported to be similar between PRKO and WT mice and our results agree with the previous findings [28], [29], [30], [31], [32]. The gonadal or reproductive hormone levels, from reproductively mature WT and PRKO mice were similar at three months of age (estrogen in the females: WT 52±13 pg/mL vs. PRKO 61±15 pg/mL; progesterone in the female: WT 7.2±3.1 ng/mL vs. PRKO 11.9±2.9 ng/mL; testosterone in the males: WT 5.6±1.0 ng/mL vs. PRKO 6.2±1.8 ng/mL). Follicle-stimulating hormone (FSH) levels were similar in the female but lower in the male PRKO mice (in females: WT 4.4 ±2.3 pg/mL, PRKO 3.9±1.6 pg/mL; in males: WT 27.7±5.3 pg/mL, PRKO 6.7±2.7 pg/mL). Inhibin A levels were higher in the female PRKO and were similar in the male mice (in females: WT 58.4±14.3 pg/mL, PRKO 169.1±38.9 pg/mL; in males: WT 50.4±6.9 pg/mL, PRKO 41.3±7.2 pg/mL).

### Mice without the nuclear PR had higher bone mass than WT mice

**Figure 1:**
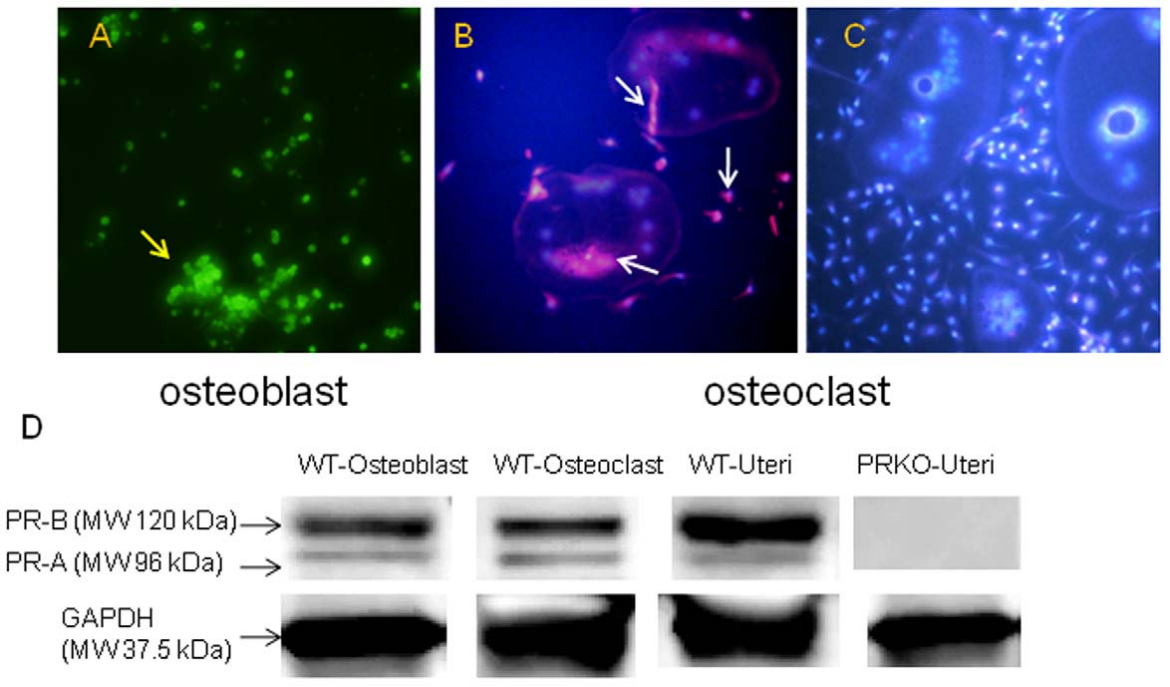
Expression of the progesterone nuclear receptor (PR-A and PR-B) in osteoblast and osteoclast. Bone marrow cells were derived from WT mice and differentiated into osteoblasts in an osteogenic medium with ascorbic acid and β-glycerophosphate or into osteoclasts with mCSF and RANKL stimulation. PR-A and PR-B were detected by immunofluoresence using PR (C19) primary antibody against both PR-A and PR-B for mouse and FITC–Conjugated secondary antibody for osteoblasts (A, yellow arrow) or Texas-red conjugated secondary antibody for osteoclasts (B, white arrows). DAPI was used to stain nucleus in the osteoclast culture (bright blue staining). C, osteoclast culture from the PRKO mice. Both PR-A and PR-B were expressed by WT osteoblast (A, yellow arrow) and the WT osteoclast (B, white arrows) but not by the PRKO osteoclast (C). In osteoclasts, the PR-A and PR-B expression was mainly observed around the nucleus, but weak expression was detected in the cytoplasm as well. Original magnification, 100×. B, Western blot analysis of the expression of PR-A (molecular weight 96 kDa) and PR-B (molecular weight about 120 kDa) proteins in osteoblasts and osteoclasts. Whole cell lysates were obtained from osteoblast or osteoclast cultures and probed with PR (C19) antibody. Uterus tissue lysates from the WT were used as a positive control while the uterus tissue lysates from the PRKO were used as a negative control. GAPDH was used as a housekeeping control. All the experiments were repeated three times.

In order to evaluate if the nPR was present in bone cells, we first examined nPR expression using western blotting on both osteoblasts and osteoclasts that were cultured from bone marrow cells. Uteri from WT or PRKO mice were used as positive or negative controls for nPR expression. We confirmed that the nPR was expressed by both osteoblasts and osteoclasts in the WT mice and that nPR expression was absent in the PRKO mice (Figure 1). We then studied the effects of the nPR on bone acquisition in experiments in which female and male PRKO mice or female WT mice were treated with a PR antagonist.

**Figure 2:**
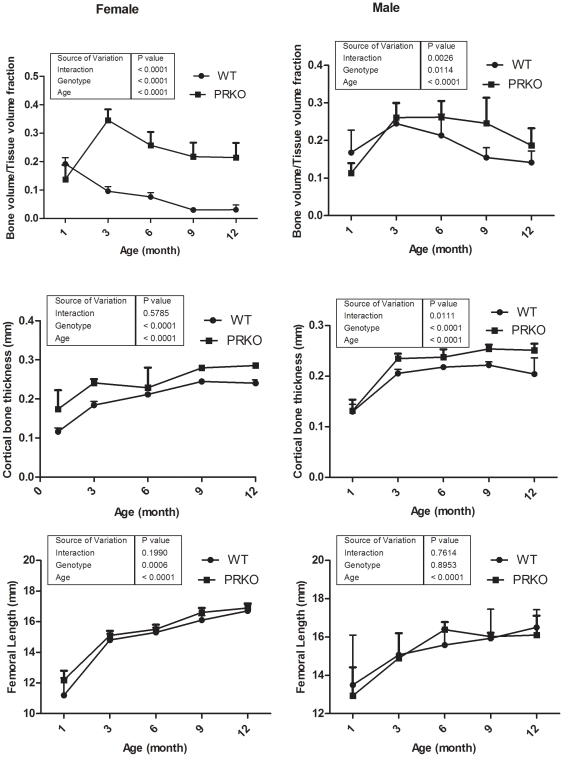
Changes in the distal femoral cancellous bone volume in the WT and PRKO mice 1 to 12 months of age. MicroCT was initiated when the mice were one-month of age and repeated at three, six, nine and 12 months on the same animals (n = 6–8 per genotype). The cancellous bone volume from the distal femur (BV/TV), cortical thickness of the mid-femur and femur length were recorded. Data was presented as mean ± SD.

The WT female mice had the highest cancellous bone mass in the distal femur between 1–3 months of age. Cancellous bone volume was lost at an average rate of 1.4% per month from 4–12 months of age. In contrast, the highest cancellous bone mass (bone volume/tissue volume ratio, BV/TV) in the distal femur was observed at three months of age, and the mass was approximately 70% higher in the PRKO female mice than the WT mice. Cancellous bone volume loss was observed at an average rate of 0.4% per month from 4–12 months of age. At 12 months of age, the female PRKO mice had 600% more cancellous bone than the WT mice (Figure 2, n = 6–8 per genotype).

In the male mice, the highest cancellous bone mass was observed in three-month-old WT mice, and bone mass was lost at an average rate of 1.1% per month between 4–12 months of age. In male, the highest femoral cancellous bone mass was observed in six-month old PRKO mice, and this cancellous bone mass was approximately 23% higher than that of the WT mice. In the PRKO male mice, cancellous bone volume loss occurred at an average rate of 0.9% per month between 3–12 months of age. The cancellous bone mass in the distal femoral metaphyses was approximately 33% higher in the male PRKO than in the WT at 12 months of age (Figure 2).

Cortical bone thickness measured at the mid-femoral shaft increased with age for both sexes and was significantly higher in the female PRKO mice than the WT mice from 3–12 months of age. There was an age-related 70% increase in cortical bone thickness in the males between 3–9 months of age, but no difference was observed between the PRKO and the WT mice. The femoral length increased from approximately 13 mm to nearly 17 mm for both WT and PRKO in both male and female mice from one to12 months of age. However, there were no significant changes in the femoral length measurements between the genotype and sexes at any of the time points (Figure 2).

### PR inhibition during linear growth period induced higher bone formation

**Figure 3:**
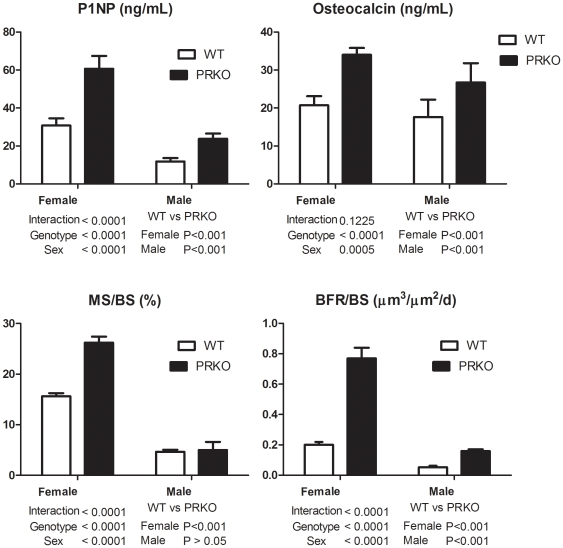
Bone formation parameters measured in the three-month-old WT and PRKO mice. Markers for bone formation, P1NP (A) and osteocalcin (B) that were measured from serum. Surfaced-based bone histomorphometry was performed at the distal femoral metaphysis that included (C), mineralized surface (MS/BS), and (D), bone formation rate/bone surface (BFR/BS). Data was presented as mean ± SD.

To examine the *in vivo* cellular activities in the PRKO female and male mice, we performed surface-based static and dynamic histomorphometry on three-month-old mice. We observed that both the surface-based bone formation parameters (mineralized surface, MS/BS and bone formation rate/bone surface, BFR/BS) and bone formation markers (propetide of type 1 procollagen (P1NP), and osteocalcin) were 50% higher in both the female and male PRKO mice than the WT mice (Figures 3, n = 8–10 per genotype) at three months of age.

**Figure 4:**
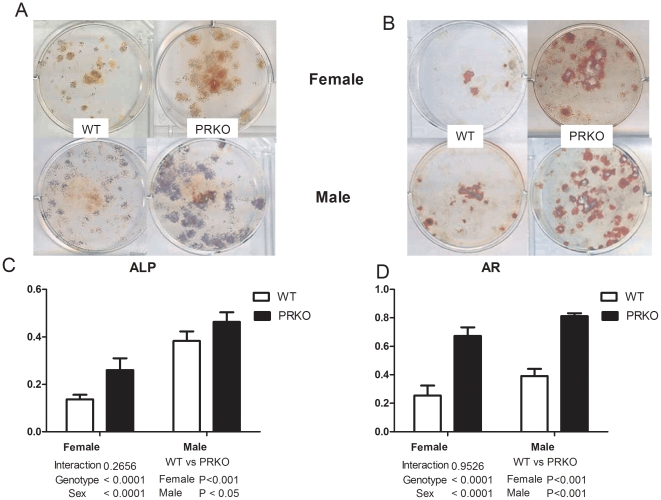
*In vitro* osteogenesis accessed in three-month-old mice. Bone marrow cells were obtained from WT female or male mice and cultured in osteogenic conditions for 14 days or 21 days and then stained for alkaline phosphatase (ALP, A and B, top pannels) or alizarin red (AR, A and B, lower pannels). The ALP level was quantified by absorbance at OD 410 nm (C)and normalized by total cell protein. The AR level was quantified by absorbance at OD 490 nm (D).

**Figure 5:**
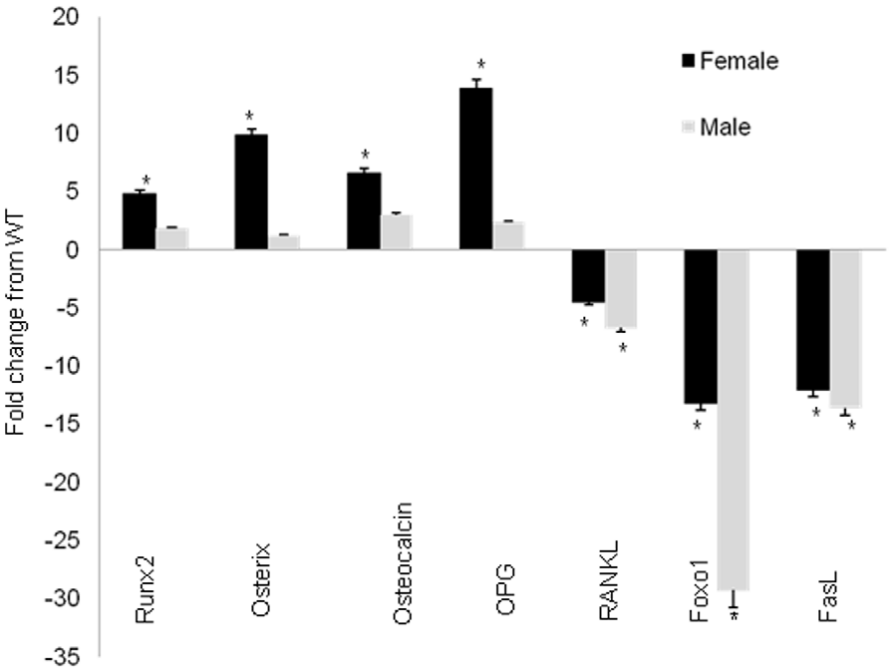
Osteoblast differentiation and apoptosis-related gene expressions in osteoblastic cultures. Bone marrow cells were extracted from 3-month-old mice and cultured with ascorbic acid and β-glycerophosphate from WT and PRKO mice to monitor osteoblastogenesis. RNA was extracted from the cultures on day 14. Real-time PCR was performed to monitor gene expression for osteoblast differentiation (Runx2, Osterix, Osteocalcin, OPG and RANKL) or apoptosis (Foxo1 and FasL). ^*^ *p*<0.05 compared with WT of the same sex.

To assess osteogenesis, we cultured osteoblasts derived from three-month-old WT or PRKO mice (n = 4–6 per genotype). Alkaline phosphatase (ALP) levels and mineralized nodule formation (stained by alizarin red, AR) were approximately 50%–100% higher in both the female and male PRKO mice compared to the WT-derived cultures (Figure 4). RNA was extracted from the osteoblast cultures and tested for expression of genes associated with osteoblast maturation, Runx2, osterix, osteocalcin and osteopontin (OPN). The levels of expression of these genes were four to 13-fold higher in the cultures from the female PRKO than the WT controls (Figure 5). Gene expression of receptor activator of NK kappa B ligand (RANKL), an osteoclast activator, was decreased by more than 4.5-fold in both female and male PRKO mice compared to the WT control mice. The expressions of genes associated with apoptosis (Foxo1 and FasL) were 15∼30-fold lower in both the female and male PRKO mice than the WT mice (Figure 5).

**Figure 6:**
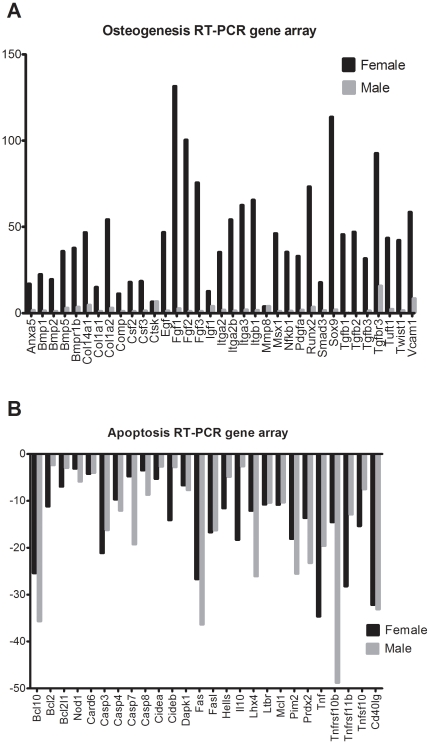
Osteogenesis and apoptosis real time RT-PCR gene arrays. RNA was extracted from the tibiae after removal of the joint and bone marrow cells from three-month-old WT and PRKO mice and run for osteogenesis real-time RT-PCR gene array (A) or real time RT-PCR apoptosis gene array (B). All the genes present in this figure were significantly differ from the WT of the same sex (*p*<0.05).

We also performed real time RT-PCR gene arrays to measure osteogenesis and apoptosis from RNA extracted from the tibial cortical bone from three-month-old WT and PRKO mice (n = 3 per genotype). The female PRKO mice had significantly higher expression of osteogenesis-related genes (Figure 6A), while both female and male PRKO mice had decreased expression of apoptosis-related genes (Figure 6B).

### PRKO mice and nPR inhibition reduced osteoclast maturation and bone resorption

**Figure 7:**
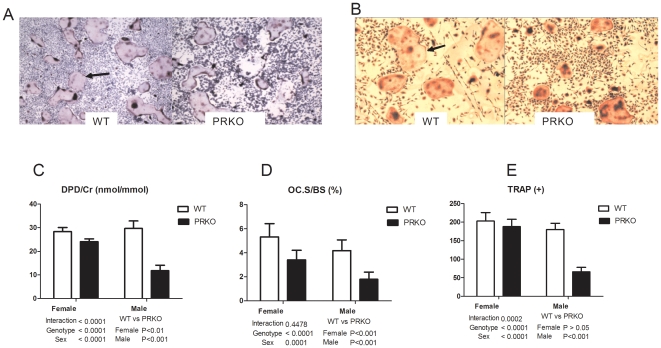
Bone resorption parameters. A, Bone marrow cells were derived from three-months old female or male mice (n = 3–4/genotype) of all genotypes and were treated in osteoclast medium that contained mCSF and RANKL for seven days. The cells were stained with TRAP to identify the osteoclasts (A and B, black arrows show TRAP+ osteoclast. Original magnification 4×). Cells with more than three nuclei were defined as TRAP+. C, qualification of the TRAP+ cells. D, DPD/Cr, was measured from the urine. E, Osteoclast surface was measured from the trabecular surface of the distal femoral metaphysis. N = 8-12/genotype for DPD/Cr and *in vivo* bone histomorphometry measurements.

Next, we studied the activity of primary osteoclast cells that were extracted from the long bones of the three-month-old mice (n =  3–5 for each genotype). We observed that mature osteoclasts, measured by TRAP+ cells, were 200% lower in male PRKO mice (Figure 7B and 7C). There was no difference between the number of TRAP+ cells in female PRKO mice (Figure 7A and 7C). *In vivo* bone resorption, measured by urinary excretion of DPD/Cr and dynamic histomorphometric measurement of the osteoclast surface, indicated that bone resorption was decreased in PRKO mice, when compared to the WT mice of the same sex (Figure 7D and 7E).

### PR inhibition by RU486 during linear growth period increased bone mass

**Figure 8:**
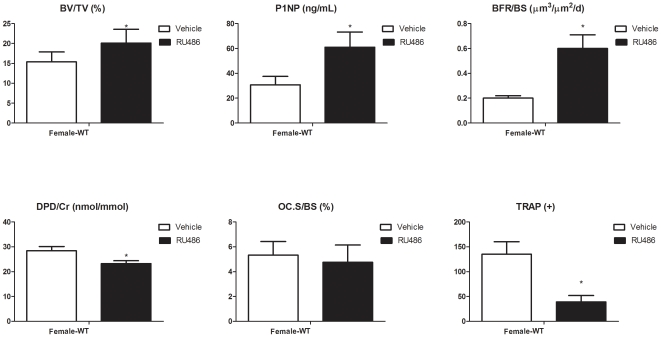
Bone mass and bone formation changes with RU486 treatment. One month-old female WT mice were treated with vehicle or RU486 (500 µg/d, 5x/week for two months). MicroCT was performed at the distal femurs to measure the cancellous bone volume (A). Bone formation was measured in either serum (P1NP, B) or at the distal femurs by surface-based bone histomorphometry (bone formation rate/bone surface, BFR/BS; C). Bone resorption was measured in the urine (DPD/Cr, D), at the distal femurs by surface-based bone histomorphometry (osteoclast surface, Oc.S/BS; E) and from bone marrow osteoclastic cultures (TRAP+, F). ^*^ *p*<0.05 compared with WT.

To determine if we could reproduce the PRKO high bone mass phenotype in female WT mice, we treated female WT mice with RU486 from one to three months of age. Cancellous bone BV/TV in the distal femur increased by 60% in female WT mice treated with RU486 compared to the WT control mice, and the BV/TV was similar to that of three-month-old PRKO mice (Figure 8). The increase in bone mass was associated with increased bone formation measured by serum bone formation markers, P1NP and surface-based bone formation rate, BFR/BS. Total osteoclast surface was not altered by RU486 treatment. However, the osteoclast maturation (TRAP+ cells formation) and function (DPD/Cr) was decreased following RU486 treatment (Figure 8).

## Discussion

We found that nPR null mice develop a higher peak bone mass when compared to the WT mice. At three months, the cancellous peak bone mass was 70% higher and the femoral cortical bone thickness was 30% greater in the female PRKO than in the WT mice. Also, the nPR null female mice demonstrated a more significant gain in bone mass during the linear bone growth period (between one to three months of age) compared to the WT controls. The male PRKO mice had 23% higher cancellous bone mass when compared to the WT when the peak bone mass was achieved at six-month of age. Both the female and male PRKO mice had slower rates of bone loss than the WT controls with age. Additionally, treatment with a progesterone antagonist, RU486, during the rapid growth period (one to three months of age) accelerated bone mass acquisition by stimulating of bone formation in the female WT mice. These data suggest that progesterone influences bone acquisition and that inhibition of nPRs during rapid bone growth can augment bone mass. Like estrogen signaling, progesterone signaling seems to have inhibitory effects on bone acquisition during the rapid bone growth period.

The pattern or bone growth and the age-related cancellous bone loss of PRKO mice were similar to previous reports [33], [34]. Rickard et al. reported female PRKO mice developed significantly higher cancellous bone mass in the tibial metaphyses than the WT mice at six and 26 weeks of age [25]. They observed that the surface-based bone formation rate was higher in six-month-old PRKO mice than the WT controls. The differences between our results and those reported by Rickard et al may be they performed their outcome measurements at different time points using different animals, whereas we followed the same groups of mice over a 12-month period. Additionally, Rickard et al. reported cancellous and cortical bone changes in the humerus and the tibiae, while we measured bone changes in the distal femurs and mid-femur. Since the trabeculae and cortical bone architecture and surface-based turnover differ between the tibia and the humeral cortical bone, this may account for the differences observed in the two studies. Despite the differences, our histomorphometric results at the distal femur were similar to their observations at the proximal tibae.

Progesterone can stimulate the proliferation and differentiation of osteoblasts in human- and rat-derived osteoblast-like cells [35], [36], [37], [38], [39], [40], [41]. In a murine osteoblast cell line culture, progesterone inhibited osteoblast apoptosis [42]. We observed that nPR (PR-A and PR-B) were present in both osteoblasts and osteoclasts. As in other studies published on the PRKO mice, we found estrogen and progesterone levels were similar between PRKO and WT mice in females and the testosterone levels were similar between the PRKO and WT mice in males [29], [31], [32]. Therefore, the PRKO bone phenotype may be due to a lack of nPR signaling rather than the changes in systemic sex hormone levels [30], [31], [32], [43], [44]. However, it is possible that the bone phenotype was induced by progesterone's non-genomic effects. To further clarify whether the PRKO bone phenotype was derived from the loss of genomic PR signaling, we treated female mice with an nPR antagonist, RU486. Female mice were used for this initial RU486 intervention study because the female PRKO mice had a significantly greater increase in bone mass than in the males. Inhibition of the nPR with RU486 in WT mice replicated the bone turnover changes and increased bone mass similar to what we observed in PRKO mice. In humans and rats [45], [46], [47], RU486 administration was reported to lower serum progesterone and increase serum estradiol, cortisol, testosterone and inhibin levels. These hormonal changes following RU486 administration were similar to what we observed in the PRKO mice. The high bone mass phenotype observed in the PRKO mice and in the WT female mice treated with RU486 was associated with increased bone formation in comparison to the WT control group.

Interestingly, we observed some sex-related differences in the bone turnover and bone cell activities between three-month-old PRKO and WT mice. In the female PRKO mice, there was accelerated bone acquisition between one to three months of age with increased osteoblastic differentiation, osteoblastic activity, and activation of the osteogenesis pathway, *in vivo* mineral apposition rate and bone formation rates. In contrast, the male PRKO mice had a modestly bone mass phenotype that developed later in their life when compared to their WT littermates. The male PRKO mice showed decreased bone resorption measured by gene expression, *in vitro* osteoclastogenesis, bone resorption marker and bone histomorphometry at three months of age. The decreased bone resorption in the male PRKO mice may be associated with the decrease in the systemic FSH level. There is some evidence that gonadotropin-releasing hormones are involved in regulating bone mass. Deletion of the FSH receptor is associated with an osteoporotic phenotype [48]. FSH directly stimulates osteoclast formation by enhancing tumor necrosis factor (TNF) production from immune cells [49]. Moreover, serum FSH increases with female reproductive aging and prior to changes in estradiol (E2) [50]. Inhibin is a gonadal hormone synthesized by the ovary and testis that can inhibit FSH production by the pituitary while activin can stimulate its release [51], [52], [53]. In females, both inhibin A and B increase during puberty and the levels correlate with the levels of estradiol and FSH in the ovary and pituitary [54]. A decrease in negative feedback from inhibin A and/or inhibin B may explain the increase in FSH with age [50], [55]. Inhibin A was recently reported to be an endocrine stimulator of bone mass and strength [56]. The systemic changes in gonadotropin-releasing hormone levels may account for the sex-related and phenotypic differences we observed in the PRKO mice.

We performed *in vitro* primary cell cultures to investigate nPR signaling during osteoblastogenesis and osteoclastogenesis in the absence of the systemic hormonal influence. *In vitro* osteoclast maturation was lower in the male PRKO mice, which suggested that nPR had intrinsic specific effects on these bone cells. Since we only measured hormonal changes and cellular activities at one time point, it is possible that we missed the “peak” cellular actions for the males. Further studies to evaluate time-dependent changes in hormone levels and cellular activities, as well as age-related changes of PR in the WT mice are necessary.

Another interesting observation was that pro-apoptotic gene expression, such as the expression of Foxo1 and FasL, was significantly lower in PRKO mice compared to WT mice. These results were measured by using an apoptosis real time RT-PCR gene array and confirmed by regular real time RT-PCR. The Forkhead O (Foxo 1, Foxo 3a, Foxo 4) subfamily of transcriptional factors are critical in cell fate decisions in response to growth factors [57], [58] and they serve as a defense mechanism against oxidative stress [57], [59], [60]. Sex hormones like estrogen and androgen have pro-apoptotic effects on osteoclasts but they also have anti-apoptotic effects on osteoblasts and osteocytes [61]. Estrogen exerts protective effects against oxidative stress in many tissues including the heart, brain, kidney and bone mainly through an anti-apoptotic mechanism [62], [63], [64], [65], [66], [67]. Only recently has estrogen's anti-apoptotic effect been linked to the inhibition of the PI3K pathway via control of Foxo 1 transcription [68]. The PR is reported to regulate the transcriptional activities of Foxos, especially Foxo1, which controls endometrial decidualization [69], [70]. A reduction in the progesterone concentration within the endometrium allows the cytoplasmic fraction of Foxo1 to enter the nucleus and bind to its direct target genes, including Bcl2 and FasL, and induce apoptosis [71], [72]. Estrogen is proposed to act through a paracrine mechanism by up-regulating FasL in osteoblasts which leads to the apoptosis of the pre-osteoclasts [73]. Testosterone and 5α-dihydrotesterone, reportedly inhibit Foxo1 and Foxo3 transcriptional activity in an androgen receptor (AR)-dependent manner [74], [75]. Androgen/AR reduces Foxo1 DNA binding and represses Foxo 1-induced cell death [76]. Our data suggests that androgens may regulate osteoblast survival via a PR-dependent mechanism. In the absence of PR, a decrease in Foxo1 and FasL transcription activities may prolong the osteoblast lifespan. FOXO proteins exist in phosphorylated and unphosphorylated forms and these alternative post-translational forms have opposing actions on anti-oxidant detoxification, DNA repair mechanisms and cellular apoptosis. Therefore, merely monitoring the overall mRNA levels of Foxo1 does not definitively assess its apoptosis capabilities. The hypothesis that PR regulates cell survival through FOXO signaling warrants additional investigation.

In addition to its classic nuclear receptor activity, some of progesterone's effects are “non-genomic” and can be explained by extranuclear signaling that involves rapid activation of Src/MAPK (mitogen-activated protein kinases), phosphoinositide 3-kinases/Akt and JaK2/Stat3 signaling pathways without the transcriptional activities of the receptors [77], [78]. The PR B-isoform is more important for activating the Src/MARK signaling pathway outside the nucleus than the A isoform of the PR [77], [79], [80]. Non-genomic progesterone actions can be initiated at the cell surface by progesterone membrane-bound receptors or through the progesterone receptor membrane component 1 (PGRMC1) [81], [82]. Activation of the MAPK pathway is the key extra-nuclear signaling pathway for steroid regulation of cell proliferation and survival in various cell types including mesenchymal-derived cells (osteoblasts and osteocytes) [83], [84], [85]. Progesterone acts directly on osteoblasts through extra-nuclear signaling and activation of G-protein coupled effectors such as phospholipase C, which leads to increased intracellular calcium and inositol trisphosphate concentrations [86], [87]. Postnatal activation of the G-proteins signaling significantly enhances osteoblast function and increases cancellous bone mass [88]. Our study with RU486 did not reproduce completely the same bone phenotype as the PRKO mice, where we observed RU486 treatment induced less bone gain compared to the RPKO, suggesting the possible involvement of progesterone extra-nuclear signaling in the regulation of bone cell activities. Progesterone extra-nuclear signaling in osteogenesis and osteoclastogenesis is currently under investigation.

This study has a number of strengths including the use of a unique PRKO mouse that lacks a nPR, and allowed the assessment of skeletal acquisition to be assessed and thoroughly characterized in the absence of this nuclear receptor. However, there are some limitations of this study. For example there is not a conditional or selective nPR knock out mouse, so we could not control for the effects of deficient nPR signaling in other tissues. Other investigators have previously reported on the reproductive and neurobiological aspects of the PRKO mice [28], [29], [30], [31], [32], [44], [89], [90]. We chose to use RU486 since it was the only pharmacological compound that was commercially available to block nPR. Because RU486 is not PR selective and it is also a partial GR antagonist, this may have confounded our observations. We are not advocating for the use of RU486 to prevent or treat osteoporosis, as it also acts as an anti-cortisol drug, which may induce an Addision-like disease with prolonged use. Another concern involving RU486 is its potential detrimental side effects, including the increased risk for endometrial cancer. However, despite the toxicity of RU486, we evaluated if a very short treatment regimen during skeletal modeling would augment bone mass. Additional studies with more selective nPR inhibitors is now warranted to further evaluate our preliminary findings.

In conclusion, mice that lack functional nPR acquire greater bone mass than WT controls. The absence of nPR is associated with increased bone formation *in vivo* and osteogenesis *in vitro* compared to age-similar controls. The high bone mass phenotype was more pronounced in the female PRKO mice than the males. Selective inhibition of the nPR with RU486 in female mice, partially reproduced the higher bone mass phenotype observed in the PRKO female mice. These data suggest that selectively inhibition of the nPR with a PR selective modular during skeletal acquisition may be a potential approach for augmenting bone mass. Further investigation of the roles of PR signaling in bone remodeling is needed.

## Materials and Methods

### Generation of the mice and experimental protocol

The PRKO breeding pairs were obtained from a research group from Dr. Judith Turgeon's laboratory at University of California, Davis. The heterozygous breeding pairs were used to generate WT, heterozygous and homozygous mice for the study. All animals were treated according to the United States Department of Agriculture (USDA) animal care guidelines with the approval of the UC Davis Committee on Animal Research. Mice were weaned at three weeks of age and genotyped using the following primers: P1, 5′-TAG ACA GTG TCT TAG ACT CGT TGT TG-3′; P3, 5′-GAT GGG CAC ATG GAT GAA ATC-3′; and N2, 5′-GCA TGC TCC AGA CTG CCT TGG GAA A-3′, which were used for genotyping in previous studies [25], [29], [32], [43].

### MicroCT measurements

Repeated *in vivo* microCT scans were performed in groups of mice (n = 6–8 per genotype) from all the genotypes between 1–12 months of age. The right distal femur and the mid-femur from each animal was scanned and measured using the VivaCT 40 (Scanco Medical, Bassersdorf, Switzerland), with a voxel resolution of 10 µm in all three spatial dimensions. We used a monoenergetic (70 KeV) X-ray sources that was reported to be safe for both structural bone endpoints and bone marrow cell viabilities in small animals [91]. The lengh of the femur was measured from a two-dimensional scout view image prior to each scan. We evaluated 200 slices, which were initiated approximately 0.2 mm away from the distal end of the growth plate. The slides covered a total metaphyses tissue volume of 2–3.5 mm^3^ for each scan and were used to obtain the cancellous bone volume/total volume (BV/TV) and cortical bone thickness at the mid-femoral region [92], [93], [94].

### Anti-nPR treatment with RU486

A separate group of one-month-old female WT mice, (n = 6), were treated with either RU486 (500 µg/mouse, subcutaneous injection, 3x/week) or vehicle (sesame oil) for two months. Mice were sacrificed at three months of age (two months post-treatment). Repeated *in vivo* microCT scans were performed at one month and three months using the same methods as above.

### Biochemical measurements

We collected serum from all the mice that we used for cell culture and bone histomorphometry to measure biochemical markers of bone formation. Serum levels of procollagen I N-terminal propeptide (P1NP) (Immunodiagnostic Systems Inc., Fountain Hills, AZ), osteocalcin (Biomedical Technology, Stoughton, MA), urine deoxypyridinoline (DPD/Cr) (Quidel Corporation, San Diego, CA) and gonadal and reproductive hormones (P, E2, FSH and inhibin A, ALPCO Diagnostics) were determined using the enzyme-linked immunosorbent assay (ELISA). The manufacturer's protocols were followed and all samples were assayed in duplicate. A standard curve was generated from each kit and the absolute concentrations were extrapolated from the standard curve. The coefficients of variations (CVs) for inter-assay and intra-assay measurements were less than 10% for all assays and were similar to the manufacturer's references [92], [93], [94].

### Bone histomorphometry

To obtain the surface based bone turnover measures, groups of three-month-old mice from all the genotypes (n = 6–8/genotype) or RU486 treated WT mice were sacrificed. These mice were injected with 20 mg/kg alizarin red and 10 mg/kg calcein seven and two days before sacrifice. The right distal femurs were removed from each mouse and fixed in neutral phosphate-buffered formaldehyde, dehydrated in graded concentration of alcohol, embedded undecalcified in methyl methacrylate and sectioned using a microtome (Leica/Jung 2255). Bone histomorphometry was performed using a semi-automatic image analysis system (Bioquant Image Analysis Corporation, Nashville, TN) linked to a microscope that was equipped with transmitted and fluorescent light. The analyses were performed in the secondary spongiosa of the distal femurs, which included the trabecular area between 100 µm to 300 µm distal to the growth plate and excluding the cortex. Bone turnover measurements included single- (sL.Pm) and double-labeled perimeter (dL.Pm), interlabel width (Ir.L.Wi), osteoclast number and perimeter. These indices were used to calculate the mineralized surface (MS/BS), mineral apposition rate (MAR), surface-based bone formation rate (BFR/BS) and osteoclast surface (Oc.S/BS) [94], [95], [96].

### Primary osteoblast culture and assays

The tibia and the left femur bone marrow cells were harvested from the femurs of three-month old WT or PRKO mice from both sexes. These bones and the serum from these mice were also used for bone histomorphometry and bone biochemical marker measurements. The cells were flushed out and plated at 3×10^6^ in 6-well plates in primary medium with phenol red free α-MEM, 10% fetal bovine serum (FBS) and 1% antibiotics. At day 5, the cells were replenished with the secondary medium containing all the ingredients of the primary medium plus 50 µg/ml ascorbic acid and 10 mM β-glycerophosphate. The cell cultures were fixed in 10% neutral buffered formalin on days 14 and 21, and each well was assayed for alkaline phosphatase (ALP) activity and mineralization (alizarin red staining). ALP activity was determined by staining the cells with a solution consisting of equal parts of p-nitrophenol phosphate (Sigma 104) and alkaline buffer solution (Sigma 221) [97]. All procedures were repeated in quadruplicate.

### Osteoclast culture

Bone marrow cells were collected from three-month-old WT or PRKO mice as described above. For the RU486 experiment, three-month-old female WT mice (n = 3/group) were treated with RU486 (500 µg/mouse, subcutaneous injection, 3x/week) for 1 week. Bone marrow cells were collected from the femurs. Cells were cultured in 24-well plates at 1×10^6^/well with 10 ng/ml macrophage colony-stimulating factor (M-CSF). After two days, non-adherent osteoclast precursors were transferred to a new plate and maintained in αMEM, 10% FBS, 101 ng/ml m-CSF, 50 ng/ml RANKL. For the tartrate-resistant acid phosphatase (TRAP) assay, the cells were stained for TRAP using the Sigma Acid Phosphatase Leukocyte Kit according to the manufacture's instructions. Multinucleated (more than three nuclei per cell) TRAP+ cells, identified by a dark purple red color, were considered mature osteoclasts. All procedures were repeated in quadruplicate.

### Western Blots/Antibodies:

Protein lysates were obtained from osteoblast or osteoclast culture in WT or PRKO mice in cold RIPA buffer. Debris was removed by centrifugation and the protein concentration was measured by the BioRad DC kit (Bio Rad). Immunoprecipitates were analyzed by SDS-PAGE and western blot analyses using standard conditions with the antibody against PR (PR C-19), Santa Cruz Biotechnology). Immune complexes were visualized following incubation with horseradish peroxidase–conjugated secondary antibody. Protein bands were detected with chemiluminescence (ECL) detection system (Amersham Biosciences). Quantification of the intensity of the bands in the autoradiograms was performed using a Kodak imaging system and analyzed by SCION IMAGE. Normalization was performed with the glyceraldehydes 3-phosphate dehydrogenase (GAPDH) antibody.

### RNA preparation and real-time RT-PCR

Total RNA was extracted from cell culture or from tibiae. After removal of the joint and bone marrow, the total RNA from bone was isolated using a modified two-step purification protocol employing homogenization (PRO250 Homogenizer, 10 mm×105 mm generator, PRO Scientific IN, Oxford CT) in Trizol (Invitrogen, Carlsbad, CA) followed by purification over a Qiagen RNeas column (Qiagen, Valencia, CA). PCRs were run 3–5 times for individual samples in each group. Real-time reverse-transcript (RT) PCR was carried out on ABI Prism 7300 (Applied Bioscience, Foster City, CA). Primer sets for real-time RT-PCRs were purchased from SABioscience (Frederick, MD). All the test genes were expressed relative to a control gene, β-actin or GAPDH. The results were expressed as fold changes from WT group, where fold changes = 2^−ΔΔCt^. Osteogenesis and apoptosis real time RT-PCR gene arrays (n = 3 per genotype) were purchased from SABioscience (Frederick, MD). There were 96 genes (wells) for each array including test genes, house keeping, no primer and no cDNA controls (detailed gene information can be found at http://www.sabiosciences.com/RTPCR.php). We excluded genes that had Ct values of 36 or more cycles as low expression levels can result in large-fold changes, but the differences were not significant. After these exclusions, 71 genes remained on the osteogenesis RT-PCR arrays, and 70 genes were left on the apoptotic PCR arrays. Gene expressions that were significantly differed from WT were presented in this report.

### Statistics

The means and standard deviations were calculated for all parameters from all groups. Repeated measures analysis of variance (ANOVA) was used to evaluate parameters derived from repeated *in-vivo* micro-CT scans such as cancellous bone volume (BV/TV), cortical thickness (Ct,Th) and femoral length and Bonferroni post-tests were used to compare time (age)-dependent changes within the same sex or between WT and PRKO at the same time point. Peak bone mass was defined as the highest BV/TV value obtained that was significantly different from baseline. For the measurements collected in three-month-old mice (bone markers, cell culture assays, bone histomorphometric parameters), two-way ANOVA was used to invest the main effects of sex, genotype and their interactions. Bonferroni post-hoc tests were used to make comparisons between the groups (SPSS Version 12, SPSS Inc., Chicago, IL; GraphPad Prism, La Jolla, CA). The real time RT-PCR findings were expressed as fold changes from the WT. Unpaired *t-test* was used to detected difference between PRKO and WT mice within the same sex. Differences were considered significant at *p*<0.05.
